# S49. EFFICACY OF HIGH-FREQUENCY REPETITIVE TRANSCRANIAL MAGNETIC STIMULATION ON PANSS FACTORS IN SCHIZOPHRENIA WITH PREDOMINANT NEGATIVE SYMPTOMS – RESULTS FROM AN EXPLORATORY RE-ANALYSIS

**DOI:** 10.1093/schbul/sby018.836

**Published:** 2018-04-01

**Authors:** Maximilian Hansbauer, Thomas Wobrock, Birgit Kunze, Berthold Langguth, Michael Landgrebe, Joachim Cordes, Wolfgang Wölwer, Georg Winterer, Wolfgang Gaebel, Göran Hajak, Christian Ohmann, Pablo Verde, Marcella Rietschel, Raees Ahmed, William Honer, Berend Malchow, Wolfgang Strube, Thomas Schneider-Axmann, Peter Falkai, Alkomiet Hasan

**Affiliations:** 1 Ludwig Maximilian University; 2 Universität Göttingen; 3 University of Regensburg; 4 Heinrich-Heine University; 5 Charite – University Medicine Berlin; 6 Sozialstiftung Bamberg; 7 European Clinical Research Network (ECRIN); 8 Central Institute of Mental Health; 9 University of British Columbia

## Abstract

**Background:**

Repetitive transcranial magnetic stimulation (rTMS) applied to the left frontal lobe is discussed to be a promising add-on treatment for negative symptoms in schizophrenia. The Positive and Negative Syndrome Scale (PANSS) has been used as outcome parameter in several previous rTMS trials, but studies focusing on PANSS factor analyses are lacking.

For this purpose, we used the available PANSS data of the ‘rTMS for the Treatment of Negative Symptoms in Schizophrenia’ (RESIS) trial to calculate different literature-based PANSS factors and to re-evaluate the impact of rTMS on negative symptoms in this trial.

**Methods:**

In an exploratory re-analysis of published data from the RESIS study (Wobrock et al. 2015), we tested the impact of rTMS applied to the left dorsolateral prefrontal cortex on two PANSS factors for negative symptoms in psychotic disorders as well as on a PANSS five-factor consensus model intending to show that active rTMS treatment improves PANSS negative symptom subscores.

**Results:**

In accordance to the original analysis, all PANSS factors showed an improvement over time in the active and, to a considerable extent, also in the sham rTMS group. However, comparing the data before and directly after the rTMS intervention, the PANSS excitement factor improved in the active rTMS group significantly more than in the sham group, but this finding did not persist if follow-up data were taken into account. These additional analyses extend the previously reported RESIS trial results showing unspecific improvements in the PANSS positive subscale in the active rTMS group.

Our PANSS factor-based approach to investigate the impact of prefrontal rTMS on different negative symptom domains confirmed no overall beneficial effect of the active compared to sham rTMS.

**Discussion:**

This secondary analysis of the RESIS trials has several limitations. First of all, the analysis of the primary endpoint was negative [24] and all subsequent secondary analyses showing a positive effect of the intervention (here: change in PANSS excitement factor) are of limited statistical power and therefore subject to uncertainty. On the other hand, our analyses confirm the negative finding of the original publication extends this finding to a broader negative symptom definition. Moreover, the new analysis provides a possible, but hypothetical explanation for the previously described effect of active rTMS on PANSS positive subscale. Of course, many other PANSS factor models are available and in pharmacological research the Marder factors [23, 35] have particular significance. However, the here used five-factor consensus model [21] includes the Marder factor results and our negative symptom factors overlaps with those factors. Another limitation is that it may be possible that our sham stimulation (coil tilted over one wing at an angle of 45°[24]) may still have been slightly biologically active as discussed elsewhere [24].

